# Metformin during Pregnancy: Effects on Offspring Development and Metabolic Function

**DOI:** 10.3389/fphar.2020.00653

**Published:** 2020-06-17

**Authors:** Gonzalo Jorquera, Bárbara Echiburú, Nicolás Crisosto, Ramón Sotomayor-Zárate, Manuel Maliqueo, Gonzalo Cruz

**Affiliations:** ^1^ Centro de Neurobiología y Fisiopatología Integrativa (CENFI), Instituto de Fisiología, Facultad de Ciencias, Universidad de Valparaíso, Valpararaíso, Chile; ^2^ Laboratory of Endocrinology and Metabolism, West Division, Faculty of Medicine, University of Chile, Santiago, Chile; ^3^ Unit of Endocrinology, Clínica Las Condes, Santiago, Chile

**Keywords:** metformin, pregnancy, polycystic ovary syndrome, diabetes mellitus, postnatal, bodyweight

## Abstract

Maternal obesity during pregnancy and gestational diabetes mellitus (GDM) are both associated with of several postnatal diseases in the offspring, including obesity, early onset hypertension, diabetes mellitus, and reproductive alterations. Metformin is an oral drug that is being evaluated to treat GDM, obesity-associated insulin resistance, and polycystic ovary syndrome (PCOS) during pregnancy. The beneficial effects of metformin on glycemia and pregnancy outcomes place it as a good alternative for its use during pregnancy. In this line of thought, improving the metabolic status of the pregnant mother by using metformin should avoid the consequences of insulin resistance on the offspring's fetal and postnatal development. However, some human and animal studies have shown that metformin during pregnancy could amplify these alterations and be associated with excessive postnatal weight gain and obesity. In this minireview, we discuss not only the clinical and experimental evidence that supports the benefits of using metformin during pregnancy but also the evidence showing a possible negative impact of this drug on the offspring's development.

## Introduction

Metformin has been one of the most successful drugs for the treatment of type 2 diabetes mellitus in the world. The pleiotropic effects of metformin have led scientists to propose its use for the treatment of other pathologies including polycystic ovary syndrome (PCOS) (Legro et al., 2013; Johnson, 2014) and cancer (Chae et al., 2016). In addition, the lack of severe adverse effects and its low cost have positioned metformin as one of the drugs with the best risk/benefit ratio.

Metformin is an orally used biguanide absorbed mainly in the small intestine through organic cation transporters (Markowicz-Piasecka et al., 2017). It enters the enterohepatic circulation arriving in the liver, which is one of its main targets, and then, it is distributed throughout the body entering the cells through organic cation transporters (Markowicz-Piasecka et al., 2017). These transporters are also expressed in the placenta, thus permitting the transport of metformin into the fetal blood. Once inside the cell, metformin inhibits the Complex I within the mitochondrion, decreasing the efficiency of the electron transport chain, leading to a decrease in ATP generation. Metformin also inhibits AMP deaminase, increasing AMP levels through this pathway. The increase in the AMP : ATP ratio activates the AMP-activated protein kinase (AMPK), a serine/threonine kinase that has a wide range of intracellular effects leading to the activation and inhibition of catabolic and anabolic pathways, respectively (Herzig and Shaw, 2018). Some of the pathways regulated by AMPK are lipogenesis, gluconeogenesis, mechanistic target of rapamycin (mTOR), and NF kappa B signaling, among others. [For a review regarding metformin mechanisms of action please see (Foretz et al., 2019)].

Recently, several studies have analyzed the use of metformin as an alternative to treat gestational diabetes mellitus (GDM), PCOS during pregnancy and even maternal obesity during pregnancy. Here, we review both the evidence supporting the use of metformin during pregnancy along with studies in humans and animal models regarding the long-term effects of metformin in the offspring.

## Use of Metformin During Pregnancy

### Gestational Obesity

Obesity during pregnancy is associated with a greater mortality and morbidity of both mother and child (Leddy et al., 2008). Obesity during pregnancy is associated with a higher risk for pregnancy loss, preterm delivery, hypertension, preeclampsia, and GDM (Lynch et al., 2008). Moreover, gestational obesity is also related to fetal macrosomia (Cedergren, 2004). On the other hand, obesity during pregnancy increases five times the risk of stillbirths and is associated with placental dysfunction (Nohr et al., 2007). Randomized, double-blind, and placebo-controlled trials, using metformin (1,000 to 3,500 mg per day starting during first trimester) have shown a slight reduction in maternal weight gain in normoglycemic obese pregnant women (Elmaraezy et al., 2017; Dodd et al., 2018; Dodd et al., 2019). However, metformin administration is accompanied with gastrointestinal side effects including nausea, diarrhea, and vomiting (Chiswick et al., 2015; Dodd et al., 2019). The risk to develop GDM is not reduced (Chiswick et al., 2015; Sales et al., 2018; Dodd et al., 2019), but one study reports lower serum levels of C-reactive protein and interleukin-6, with a significant reduction in the risk to develop preeclampsia (Syngelaki et al., 2016). Yet, these effects are not associated with an improvement in insulin sensitivity (Balani et al., 2017). Metformin does not reduce the incidence of large for gestational age newborns, nevertheless, it reduces the risk of neonatal intensive care unit admissions (Chiswick et al., 2015; Elmaraezy et al., 2017; D'Ambrosio et al., 2019; Dodd et al., 2019). Therefore, the beneficial effects of metformin on maternal and neonatal health when used in gestational obesity are still under debate and its prescription should be cautious.

### Gestational Diabetes

The prevalence of GDM varies between 10% and 20% according to the diagnostic criteria, ethnicity, and personal and family history (DeSisto et al., 2014; Chiefari et al., 2017; Bashir et al., 2018). Approximately 1 out of 7 pregnancies is affected (International Diabetes Federation, 2017; Chow et al., 2018). GDM is associated with short- and long-term sequelae on both, mother and offspring (Dall et al., 2014; Farrar et al., 2017). Dietary intervention is the primary treatment (Crowther et al., 2005; Landon et al., 2009) and insulin is the classical pharmacotherapy for GDM. However, metformin has gained wide acceptance and has been recommended as a safe alternative to insulin for the treatment of GDM (Hod et al., 2015) (Guidelines N. Diabetes in Pregnancy. NICE Guide 2015. https://www.nice.org.uk/guidance/ng3). The Society for Maternal-Fetal Medicine (SMFM) recently published that in women with GDM in which hyperglycemia cannot be managed by medical nutrition therapy, metformin is a reasonable and safe first-line pharmacologic alternative to insulin. (SMFM Publications Committee, 2018).

Several studies showed reduced weight gain in women with GDM treated with metformin vs. those treated with insulin, along with a reduction of severe hypoglycemia and pregnancy-induced hypertension (PIH) (Rowan et al., 2008; Alqudah et al., 2018). A systematic review and meta-analysis revealed that metformin is better than insulin in reducing both, maternal weight gain during pregnancy and the frequency of PIH, with no changes in the frequency of hypoglycemia and preeclampsia (Gui et al., 2013). In addition, recent randomized controlled trials (RCT) suggest that metformin could be used to treat or prevent preeclampsia (Romero et al., 2017). Interestingly, the Metformin in Gestational Diabetes (MiG) study showed a higher acceptability for metformin than insulin, despite the gastrointestinal side effects produced by metformin (Rowan et al., 2008). However, metformin has a high percentage of ineffectiveness in the management of glycemia in GDM patients and 46% of women in metformin group needed to incorporate insulin into their treatment (Rowan et al., 2008). A study in a retrospective cohort of Qatar women with GDM observed that metformin in comparison with nutritional therapy, reduces some adverse effects of GDM on pregnancy outcomes, such as maternal weight gain, risk of macrosomia, and neonatal hypoglycemia (Bashir et al., 2018). It is important to note that metformin has been proposed as a safe medication without teratogenic effects (Ainuddin et al., 2015; Jiang et al., 2015; Given et al., 2018). To date, several studies have shown that metformin therapy has greater benefits compared with insulin and glibenclamide as GDM treatments, such as lower risk of large for gestational age babies or macrosomia, neonatal hypoglycemia, and admission to neonatal intensive care units (Rowan et al., 2008; Butalia et al., 2017; Farrar et al., 2017). Although others have observed deleterious effects of metformin, such as an increased rate of preterm birth (Rowan et al., 2008; Su and Wang, 2014). Nevertheless, a recent systematic review shows an absence of metformin associated increase in the rate of preterm delivery, caesarean section or risk of small for gestational age babies (Butalia et al., 2017).

Metformin use has shown more favorable pregnancy outcomes when compared to a controlled diet, but the efficacy when compared to the effects of insulin, is still under debate. While a meta-analysis of six RCT concluding that metformin use in GDM is not significantly associated with adverse maternal or neonatal outcomes (Su and Wang, 2014), the evidence also fails to confirm a higher beneficial effect of metformin compared to the use of insulin. Although the use of metformin in pregnancy is not supported by many international clinical guidelines, its use has increased recently (Rowan et al., 2008; Priya and Kalra, 2018). Recommendations regarding metformin use in pregnancy vary widely, partly because of its ability to cross the placenta (Simmons et al., 2004; Hyer et al., 2018; Simeonova-Krstevska et al., 2018). While some studies found no clear evidence of any adverse outcomes related to the use of metformin for the treatment of GDM in pregnancy (Priya and Kalra, 2018; Maple-Brown et al., 2019), others have reported comparable outcomes to insulin therapy (Rowan et al., 2008). Many medical associations have suggested that insulin should be considered as a first-line agent for managing GDM (Lindsay and Loeken, 2017), along with appropriate lifestyle changes (Lindsay and Loeken, 2017). Despite the considerable evidence suggesting that metformin is safe during pregnancy, long-term safety data are still needed (Simmons et al., 2004; Hyer et al., 2018; Simeonova-Krstevska et al., 2018).

### Polycystic Ovary Syndrome

PCOS is a highly prevalent endocrine-metabolic dysfunction (5-10% of women in reproductive age) which is associated with metabolic disturbances that have a high impact in cardiometabolic diseases, such as insulin resistance (Dunaif et al., 1989; Franks, 1995; Holte, 1996) and pancreatic beta cell dysfunction with subsequent development of type 2 diabetes (O'Meara et al., 1993; Ehrmann et al., 1999; Legro et al., 1999). PCOS patients—diagnosed according to the NIH criteria (presence of hyperandrogenism and chronic anovulation) (Zawadski and Dunaif, 1992)—have an increased metabolic risk. In this group the prevalence of glucose intolerance is 23% to 35% and 4% to 10% for type 2 diabetes (T2D) (O'Meara et al., 1993; Ehrmann et al., 1999; Legro et al., 1999; Ehrmann et al., 2005). According to the Rotterdam definition, the diagnosis of PCOS may be established with at least 2 out of 3 criteria: hyperandrogenism, oligo-anovulation, and a polycystic ovary on ultrasound image (Group R. E. A. S. P. C. W., 2004), generating four different phenotypes. Hyperandrogenism is the main factor for the development of metabolic and cardiovascular alterations (Azziz et al., 2009; Fauser et al., 2012).

PCOS patients have an increased risk for pregnancy complications. A 2011 a meta-analysis shows that the odds ratio for different pregnancy complications is significantly increased in PCOS patients compared to controls. Most recently, Palomba et al. found a 3- to 4-fold increase in PIH and preeclampsia, 3-fold increase in GDM, and a 2-fold higher chance for premature delivery (Palomba et al., 2015) among PCOS patients. Authors concluded that characteristics associated to PCOS, such as hyperandrogenism, obesity, insulin resistance, and other metabolic abnormalities, may contribute to the increased risk of obstetric and neonatal complications (Palomba et al., 2015). Women with PCOS have shown placental inflammation, placental thrombosis and infarction during pregnancy, added to villous immaturity and nucleated fetal red blood cells (Koster et al., 2015). These alterations may be signs of vascular damage and fetal hypoxia which could be linked to complications in PCOS pregnancies (Koster et al., 2015).

Metformin has been evaluated in several studies in the context of PCOS pregnancy. It has been reported an 18.7% incidence of miscarriage in non-treated PCOS women, 10% in women who received metformin up to 32 weeks of gestation, and 0% in those who received metformin throughout pregnancy (p < 0.006) (Nawaz et al. 2008). A 2012 meta-analysis reports that in five out of seven studies analyzed, metformin reduces the development of GDM in pregnant women with PCOS (Ghazeeri et al 2012). As the methodologies were different and there were no RCT at that time, these results are inconclusive. The biggest and more accurate RCT regarding this issue was performed by Vanky et al. Their work showed no effect of metformin on the prevalence of GDM and preeclampsia and only a significant reduction in preterm delivery in a subgroup of compliant patients in the per protocol analysis (Vanky et al., 2010). A second RCT from the same group reported a reduction in the risk of late miscarriage and preterm birth with no difference in the prevalence of GDM (Lovvik et al., 2019). The effects of metformin on preterm delivery are of notable interest given the known association of preterm delivery with neonatal mortality, respiratory distress syndrome, cerebral palsy, gastrointestinal and cardiovascular disorders, retinopathy of prematurity, high cost related to prenatal care, among many other consequences (Behrman and Butler, 2007). Indeed, some clinical trials have started to evaluate the effect of metformin to avoid preterm delivery in patients with preeclampsia (Cluver et al., 2019).

We compared the effects of metformin treatment in a historic PCOS group and compared them to untreated patients in a small cohort study. Our results show that the prevalence of GDM drops from 36% to 14% (Crisosto et al., 2012). This study was designed to evaluate differences in the offspring of PCOS patients treated and not treated with metformin. Our untreated patient group had an important pathogenic profile, with increased androgen and insulin levels. Metformin use normalized these parameters, and the female offspring of our patients showed a normalization of AMH levels, a marker illustrating the prevention of ovarian programing. We think metformin worked very well because our PCOS patients had a 36% prevalence of GDM. Nevertheless, in the first Vanky study (Vanky et al., 2010) untreated patients had the same basal prevalence of our treated group. In that setting, it is difficult to see an effect. Therefore, the usefulness of metformin is not a black and white issue, it depends on the profile and the phenotype of the patient.

## Postnatal Effects of Metformin Exposure During Prenatal Life

### Evidence in Humans

#### Postnatal Effects of Metformin in a PCOS Context

Few reports have explored the postnatal effects of metformin in humans. These studies have been carried out in children of different ages born to mothers with PCOS or GDM during pregnancy, hence comparisons among these results should be done cautiously. The first studies by Glueck et al. in a PCOS US population, did not show differences in growth and motor–social development after 3, 6, 9, 12, and 18 months after birth in children whose mothers were exposed to 1.5 to 2.55 g/day of metformin during pregnancy (Glueck et al., 2014).

We published that metformin use is able to normalize androgens and insulin levels during pregnancy, and reduce AMH levels, an ovarian marker associated to a higher risk of PCOS in the female offspring as early as 2 two months of life (Crisosto et al., 2012). Lately, studies carried out in Norwegian children born to women with PCOS receiving 2.0 g of metformin daily from the first trimester to delivery, show that women who used metformin in pregnancy lost less weight and their infants were heavier than those in the placebo group one year postpartum (10.2 ± 1.2 kg vs 9.7 ± 1.1 kg) (Carlsen et al., 2012). Recently, the same researchers observed that 1-year old females that had been exposed to metformin in utero had a larger head circumference compared to non-exposed girls, a feature not observed in boys. At 4 years of age, both males and females maintain a higher weight with an odds ratio of 2.17 (1.04–4.61) to be overweight/obese (Hanem et al., 2018). Another study in women with PCOS treated with 850 mg of metformin from the first trimester to term, shows that, at 8 years of age, children exposed prenatally to metformin exhibits higher glucose levels and a strong trend towards elevated systolic blood pressure and a lower LDL cholesterol level (Ro et al., 2012).

#### Postnatal Effects of Metformin in a GDM Context

In GDM, children at 18 month of age and exposed to metformin are heavier and taller than those exposed to insulin during pregnancy. However, motor, social, or linguistic development did not differ between the groups (Ijas et al., 2015). At 2 years of age, children of the metformin group present with a larger skinfold (mid-upper arm circumferences, subscapular, and biceps skinfold), although fat mass and body fat percentage, assessed by bioimpedance and DEXA, are not different (Rowan et al., 2011; Wouldes et al., 2016). Moreover, in these children, no differences in blood pressure and neurodevelopmental tests are reported (Battin et al., 2015). By 9 years of age, children exposed to metformin evidence larger waist circumferences, waist/height ratio, BMI, and triceps skinfold. Interestingly, fat mass evaluated by DEXA and MRI tends to be higher in the metformin compared to the insulin group, suggesting a higher risk for development metabolic syndrome but with comparable biochemical parameters (Rowan et al., 2018). On the other hand, a study including 1,996 children born to women with GDM treated with metformin and 1,932 treated with insulin shows no differences in child growth and neurodevelopment between both groups (Landi et al., 2019). Also, one study compares the testicular size in prepubertal boys born to mothers who participated in a RCT contrasting metformin with insulin in the treatment of GDM. No differences in testicular size were found from 33 to 85 months of age (Tertti et al., 2016). Finally, a meta-analysis including 10 randomized studies in women with PCOS or GDM with a total of 778 children and a maximal follow-up duration of 9 years shows that children exposed in utero to metformin are heavier than controls, although the BMI z-score are comparable between groups (van Weelden et al., 2018).

### Evidence from Animal Models

Recent studies have analyzed the long-term metabolic effects linked to metformin exposure during prenatal life in animal models. In one of these studies murine dams under regular diet are treated with 300 mg/kg/day during pregnancy (Salomäki et al., 2013). It finds that intrauterine metformin exposed females, but not males, fed with a regular diet have higher body weight between 4.5 and 7 weeks of age. When the offspring exposed to metformin are fed with a high fat diet (HFD) for 8 weeks, both males and females (at 17 weeks old), demonstrate higher weight gain compared to the offspring of mothers not treated with the drug. The authors also show elevated fasting glucose levels and an impaired glucose tolerance test in males fed with HFD exposed to metformin in prenatal life (Salomäki et al., 2013).

In another article, female mice are fed with a HFD 1 month before mating and during the gestation period and with or without oral administration of metformin 300/mg/day during pregnancy (Salomäki et al., 2014). During the second week of gestation metformin-treated HFD dams exhibit diminished fasting glucose levels compared to those treated with vehicle. Fetuses exposed to metformin are lighter at E18.5 compared to those not exposed. At 10 to 11 weeks old, offspring started a HFD until week 17. Intrauterine exposure of females to metformin and HFD, results in less body weight gain and reduced percentage of fat along with an increased percentage of lean mass compared not exposed prenatally females (Salomäki et al., 2014). In addition, the female offspring of metformin-treated dams present with less weight in all the white adipose tissue (WAT) deposits. In glucose tolerance tests, HFD animals from both genders present with improved glycemic responses when their mothers received metformin during pregnancy (Salomäki et al., 2014). Similar results are observed in a study where pregnant mice have access, during the whole pregnancy, to regular diet and water in which metformin is disolved (5 mg/ml) (Gregg et al., 2018). Young 6-week-old males exposed prenatally to metformin show a better glucose management during an intraperitoneal glucose tolerance test, and they secrete more insulin after glucose injection compared to controls (Gregg et al., 2018). In females exposed in utero to metformin, there were no changes in metabolic parameters at young ages, but at 1 to 2 years old, they have a better glucose tolerance response. Finally, these authors have shown that β-cells from males exposed to metformin secrete more insulin after high glucose (22 mM) or KCl (30 mM) stimulation in an *in vitro* assay (Gregg et al., 2018).

A different article studied the ability of prenatal metformin treatment to diminish signs of metabolic syndrome in the offspring (Tain et al., 2018). Rat dams received a diet high in fructose during the gestation and lactation periods. A group of mothers were treated with metformin 500 mg/kg/day by gastric gavage. Only male offspring were selected, because males are prone to develop hypertension compared to females. Male offspring were fed with control or high fat/high sucrose diet (HFS) after weaning. Twelve-week-old males fed with HFS developed hypertension with an increase of 15 mm Hg in mean arterial pressure compared to controls, and the increase in blood pressure was prevented by prenatal metformin exposure (Tain et al., 2018).

We published a study in which HFD fed pregnant rats had access to water in which metformin was dissolved, 1 week before mating, during pregnancy and nursing (Álvarez et al., 2018). Doses were estimated at 160 to 200 mg/kg/day. We found that body weights of the female offspring exposed to metformin were higher compared to those not exposed, from day 1 to day 60 of age (Álvarez et al., 2018). This difference disappeared after day 60. Females exposed to metformin also had increased retroperitoneal WAT depots, hyperleptinemia, and a tendency to hyperinsulinemia at 2 months old (Álvarez et al., 2018).

Other studies have explored the reproductive function in the offspring of mothers treated with metformin during pregnancy. Recent research has established that prenatal exposure to metformin (300 mg/kg/day to pregnant mice dams) induces a reduction in testicular size in fetal and neonatal states with a significant reduction in the Sertoli cell population (Tartarin et al., 2012). At long term, exposure to metformin during gestation and lactation induces a reduction in sperm count in male rats at 110 days after birth (Forcato et al., 2017). In addition, we observed that metformin treatment (1 week before mating until weaning) in HFD-fed rat dams improved ovarian function in the adult female offspring (Álvarez et al., 2018). Moreover, our study showed that prenatal metformin treatment was not able to prevent early puberty onset, induced by an in utero obesogenic environment. However, the treatment reduced the number of antral follicles, follicular cysts, and multi-oocyte follicles in the ovaries of the adult female offspring of obese mothers treated with metformin, which could be useful for diminishing the risk of PCOS development (Álvarez et al., 2018).

## Concluding Remarks

The use of metformin as a first-line drug during pregnancy is still controversial. However, it is important to note that the specific features of each patient (PCOS, hyperandrogenism, GDM, low adherence to insulin, risk of preeclampsia) are important to consider when estimating the risk/benefits ratio. The beneficial effects of metformin on pregnancy outcomes are clear in GDM and PCOS pregnancies with insulin resistance. However, the postnatal long-term effects require more evidence. Metformin appears to prevent ovarian developmental programming, but it could be associated to an increased bodyweight and metabolic derangements both in humans and rodents. In this context, the use of metformin during pregnancy has to be analized according to the risk/benefit ratio on each specific group of patients, while waiting for better evidences.

## Author Contributions

GJ and RS-Z reviewed data in animal models. BE, NC, and MM reviewed data of human studies. GC coordinated and organized the entire manuscript. GJ and GC edited the final version of the manuscript.

## Funding

This work was supported by Centro de Neurobiología y Fisiopatología Integrativa (CENFI), Universidad de Valparaíso. Grant DIUV-CI 01/2006.

## Conflict of Interest

The authors declare that the research was conducted in the absence of any commercial or financial relationships that could be construed as a potential conflict of interest.
